# Efficacy and safety of tislelizumab plus bacillus-calmette guérin with or without chemotherapy as a bladder-sparing treatment for high-risk non-muscle-invasive bladder urothelial cancer: a real-world study

**DOI:** 10.1007/s12672-024-01146-2

**Published:** 2024-07-08

**Authors:** Peng Wu, Wei Zhang, Wei Hu, Yitong Cao, Jia Wang, Lei Yu

**Affiliations:** 1 Department of Urology, Xijing Hospital, Air Force Medical University, No. 127 Western Changle Road, Xi’an, Shaanxi, 710032 P.R. China; 2 Department of Endocrinology, Xi’an No. 3 Hospital, the Affiliated Hospital of Northwest University, Xi’an, Shaanxi 710018, P.R. China

**Keywords:** High-risk non-muscle-invasive bladder cancer, Tislelizumab, Bacillus-calmette guérin, Efficacy, Safety

## Abstract

**Background:**

Despite adequate transurethral resection of the bladder tumor (TURBT) followed by intravesical bacillus-calmette guérin (BCG), high-risk non-muscle-invasive bladder cancer (HR-NMIBC) is associated with high rates of recurrence and progression. Immune checkpoint inhibitors can improve antitumor activity in bladder cancer, but relevant evidence in HR-NMIBC is limited. Thus, we evaluated the efficacy and safety of the tislelizumab-based combination regimen in HR-NMIBC.

**Methods:**

A retrospective study included 21 patients diagnosed with HR-NMIBC between July 2020 and September 2022. All patients underwent TURBT followed by combination regimens of tislelizumab plus BCG with or without gemcitabine/cisplatin (GC) chemotherapy. Clinical Data on demographics and characteristics, treatment information, outcomes, and safety were collected and analyzed.

**Results:**

Among the 21 patients with HR-NMIBC, the median age was 63 years (range 39–85), with the majority of patients with stage T1 (16/21, 76.19%). The median treatment of tislelizumab was 5 cycles (range 1–12) and the median number of BCG instillations was 12 times (range 2–19). Of the 21 patients, 15 (71.43%) received combination chemotherapy with GC, with a median treatment of 2 cycles (range 0–7); others did not. Overall, after the median follow-up of 25 months (range 7–31), the estimated 2-year bladder recurrence-free survival rate was 78.64% (95% confidence intervals [CIs], 50.79–91.83%), 2-year cystectomy-free survival rate was 83.00% (95% CI 53.53–94.59%), and 2-year disease-free survival rate was 73.39% (95% CI 46.14–88.36%). Sixteen stage T1 patients achieved a distant metastasis-free survival rate of 95.45% (95% CI 71.87–99.34%) at 2 years. Fourteen (66.67%) patients experienced at least one treatment related-AEs (TRAEs), with 9.52% (2/21) of grade 3–4. Grade ≥ 3 TRAEs were hypophysitis (1/21, 4.76%) and myasthenia (1/21, 4.76%). No treatment-related deaths were observed.

**Conclusions:**

The study demonstrated promising clinical benefits and a manageable safety profile of tislelizumab-based combination regimen as a bladder-sparing treatment of HR-NMIBC.

## Introduction

Addressing high-risk NMIBC (HR-NMIBC), a subgroup characterized by a heightened risk of disease recurrence and progression, remains one of the most daunting challenges in urologic oncology [1]. There is currently no established guideline for HR-NMIBC, and conservative treatment with transurethral resection of bladder tumor (TURBT) and postoperative intravesical instillations with Bacillus-Calmette Guérin (BCG) is recommended, with complete response rates reaching approximately 75% [2]. Unfortunately, around half of the patients undergoing this regimen will encounter a recurrence within a five-year period; and approximately 21% of these cases will advance to muscle-invasive bladder cancer, accompanied by survival rates of only 35% [1, 3]. The remains 20–30% will develop incurable metastatic disease [1, 4]. For those populations, radical cystectomy (RC) is advised [2]. However, RC, while effective, is linked with much morbidity and mortality, and it can notably impact the quality of life. Consequently, many patients opt to decline this procedure [5]. Meanwhile, a considerable number of patients are unable to radical cystectomy due to factors such as advanced age or underlying chronic medical conditions [6]. Consequently, there is an urgent need to develop alternative bladder-preserving strategies to cater to the specific requirements of HR-NMIBC populations and enhance prognosis.

Currently, anti-programmed cell death protein 1 (PD-1)/programmed death-ligand 1 (PD-L1) immune checkpoint inhibition (ICI) have been have utilized for BCG-unresponsive patients who refuse or are ineligible for RC, due to their strong rationale. Based on the KEYNOTE-057 phase II trial, pembrolizumab monotherapy was approved for patients with BCG-unresponsive HR-NMIBC, which demonstrated promising antitumor activity, with 41% of patients achieving a complete response (CR) at 3 months and a median duration of response of 16.2 months [7]. In addition, due to combining PD-1 inhibitors with platinum-based chemotherapy may enhance tumor immunogenicity [8], multiple ongoing clinical trials are evaluating the efficacy of combining PD-1 with platinum-based chemotherapy (such as gemcitabine/cisplatin [GC]) in NMIBC [9]. However, given the majority of studies are ongoing and the limited clinical benefits of the published studies, the optimal clinical management for HR-NMIBC patients (especially combination therapies) has not been unraveled yet [10].

Tislelizumab (BeiGene, Co., Ltd., Beijing, China), a humanized IgG4 monoclonal antibody, exhibits high affinity and binding specificity for PD-1. Unlike other antibodies, it minimizes Fcγ receptor binding on macrophages, thereby disrupting antibody-dependent phagocytosis-a mechanism involved in T cell clearance and potential resistance to anti-PD-1 therapy [11, 12]. Compared to pembrolizumab, tislelizumab demonstrates a higher affinity to PD-1, characterized by a 100-fold slower off-rate than pembrolizumab [13]. Recent trials assessing the efficacy of tislelizumab combined with platinum-based chemotherapy have indicated promising antitumor activity with manageable tolerability in patients with advanced non-small cell lung cancer, esophageal squamous cell carcinoma, or gastric/gastroesophageal junction adenocarcinoma [14, 15]. However, the available evidence of tislelizumab and chemotherapy for in HR-NMIBC is limited. Thus, we conducted a retrospective study to determine the efficacy and safety of tislelizumab and BCG with or without GC chemotherapy in patients with HR-NMIBC.

## Materials and methods

### Patients

This retrospective study included 21 patients with HR-NMIBC who received a first-line regimen of tislelizumab and BCG with and without GC between July 2020 and September 2022 at urologic surgery, Xijing Hospital of Air Force Military Medical University Center. The inclusion criteria were as follows: patients had pathologically confirmed urothelial cancers with or without carcinoma in situ (CIS); a strong preference bladder preservation; an Eastern Cooperative Oncology Group performance status (ECOG PS) of 0–1; with clinical stage Ta-T1N0M0; had at least one of the following high-risk factors: T1 tumors, G3 tumors, multiple tumors, recurrent tumors, CIS, Ta G1/G2 tumor diameter of > 3 cm, or variant histology subtypes. Patients with contraindications to immunotherapy or chemotherapy were excluded. This study was approved by the ethical review board of the Xijing Hospital and was conducted under the Declaration of Helsinki and local applicable regulatory guidelines. As this study was retrospective in nature, the requirement for informed consent from patients was waived by Xijing Hospital.

### Treatments

All patients first underwent maximum TURBT and received tislelizumab and BCG with or without GC chemotherapy 6 weeks later. Tislelizumab was given 200 mg intravenously on day 1 for 21-day cycles. BCG intravesical instillation was performed within 2–4 weeks after surgery, involving every week administrations initially for 6 weeks, followed by 2-weekly BCG intensified instillation for 6 weeks, and concluding with every month BCG maintenance installations for 10 months. GC chemotherapy was given in 21-day cycles, including gemcitabine (1000 mg/m^2^ intravenously, days 1 and 8) and cisplatin (70 mg/m^2^ intravenously, day 2). Patients who were intolerant to cisplatin were administered the monotherapy regimen of tislelizumab; These patients met one or more of the following criteria: renal insufficiency (eGFR ≥ 30 mL/min and < 60 mL/min), general condition with ECOG PS of 0–2, hearing loss, or grade ≥ 2 peripheral neuropathy.

During every 3-month follow-up period, TURBT was performed if patients had low-grade recurrent NMIBC; salvage cystectomy was conducted if high-grade recurrent NMIBC was present; referred to the metastatic bladder cancer management if distant metastasis was identified.

### Data collection and outcomes

All data was retrospectively collected from medical records, including baseline characteristics, tumor biological features, treatment information, clinical outcomes, and adverse events (AEs). The study outcomes were bladder recurrence-free survival (BRFS), cystectomy-free survival (CFS), disease-free survival (DFS), and distant metastasis-free survival (DMFS) for stage T1 patients. BRFS was defined as the time between the initial TURBT and the date of the TURBT for bladder recurrence; CFS was calculated from the time to the first cystectomy or death from any cause; DFS was calculated from the time between the date of TURBT and death or the development of local recurrence or distant metastasis; DMFS was defined as time of diagnosis until occurrence of distant metastasis (Recurrences cephalad to the iliac bifurcation or within the inguinal nodes were scored as distant metastases). Efficacy was evaluated using Response Evaluation Criteria in Solid Tumors version 1.1 (RECIST v1.1) with computed tomography (CT) or magnetic resonance imaging (MRI) per week during the treatment and at every follow-up visit. Meanwhile, cystoscopy and urinary cytology were performed during the follow-up period. Adverse events (AEs) including treatment and immune-related were defined and graded according to Common Terminology Criteria for Adverse Events v4.03. AEs were recorded per week during the treatment and at every follow-up visit.

### Statistical analysis

Categorical data were expressed as numbers with percentages and continuous data were expressed as median with range (minimum to maximum). Survival curves for BRFS, CFS, DFS, and DMFS were plotted using the Kaplan–Meier method. The 95% confidence intervals (CIs) for these outcomes were also calculated using the Kaplan–Meier method. A two-sided P-value < 0.05 indicated statistically significant. All statistical analysis was done using SPSS software v23.0 (SPSS Inc., Chicago, IL, USA) and graphing was performed using GraphPad Prism v8.0.

## Results

### Patient characteristics and treatment

Among the 21 patients diagnosed with HR-NMIBC, the median age of 63 years (range 39–85), with 15 (71.43%) being male. Most patients had an ECOG PS of 0–1 (20/21, 95.24%), and T1 (16/21, 76.19%) disease. The high-risk features identified that 13 patients (61.90%) with G3 high-grade tumors (including 1 had concomitant carcinoma in situ), 4 (19.05%) with multifocal tumors, 2 (9.52%) with highly recurrent tumors, and 2 (9.52%) had a tumor with mixed histology. Detailed clinical characteristics were summarized in Table 1.

**Table 1:** Baseline characteristics

Variable	Overall (n = 21)
Gender	
Male	15 (71.43)
Female	6 (28.57)
Age (years)	
Median (range)	63 (39–85)
ECOG PS	
0–1	20 (95.24)
2	1 (4.76)
T stage	
Ta	5 (23.81)
T1	16 (76.19)
Pathological subtypes	
Non-invasive papillary urothelial carcinoma	
High-grade	16 (76.19)
Low-grade	2 (9.52)
High-grade papillary urothelial carcinoma with glandular differentiation	2 (9.52)
High-grade papillary urothelial carcinoma with carcinoma in situ	1 (4.76)
High-risk features	
G3 high-grade tumors	13 (61.90)
Concomitant carcinoma in situ	1 (4.76)
Multifocal tumors	4 (19.05)
Highly recurrent tumors	2 (9.52)
Mixed histology tumors	2 (9.52)

ECOG PS, eastern cooperative oncology group performance status

In the cohort of 21 treated patients, the median treatment of tislelizumab was 5 cycles (range 1–12). Of these, 15 (71.43%) received combination chemotherapy with GC, with a median treatment of 2 cycles (range 0–7); others (6/21; 28.57%) did not underwent chemotherapy. The median number of BCG instillations for all patients was 12 times (range 2–19).

### Clinical outcomes

As of October 2023, the median follow-up was 25 months (range 7–31). Among the 21 patients, 16 achieved stable disease; 1 experienced recurrence of low-grade bladder tumor, and underwent a second TURBT and continued with postoperative maintenance treatment of BCG instillation; 2 had recurrence of high-grade bladder tumors and underwent salvage cystectomy; 1 developed distant metastasis but without progression in the bladder, subsequently initiating treatment for metastatic bladder cancer; 1 died.

**Fig. 1:**
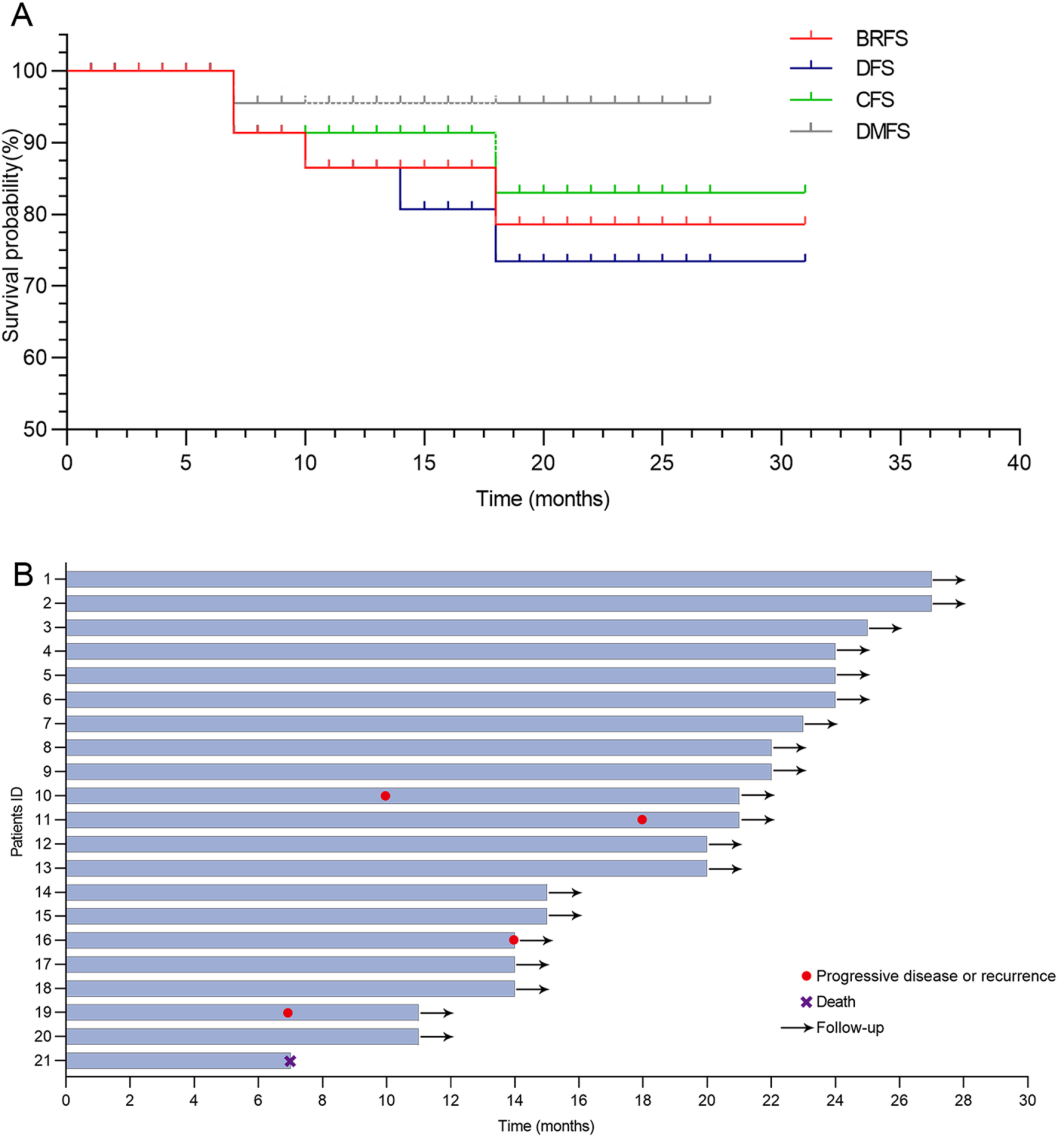
Duration of response from initiation of combination therapy. **A** Survival curve of patients with high-risk non-muscle-invasive bladder cancer receiving tislelizumab and BCG with or without GC chemotherapy as a bladder-sparing treatment strategy; **B** Time to recurrence or progression of patients with high-risk non-muscle-invasive bladder cancer. The patient 10 experienced a recurrence of non-muscle invasive bladder cancer 10 months after treatment. Then, management of TURBT, immunotherapy, and BCG instillation were performed; The patient 11 experienced a recurrence of muscle-invasive bladder cancer 18 months post-treatment and received intervention involved radical cystectomy surgery followed by postoperative adjuvant therapy; The patient 16 developed metastasis 14 months after treatment and subsequently underwent advanced systemic therapy; The patient 19 experienced a recurrence of muscle-invasive bladder cancer 7 months after treatment and received radical cystectomy surgery followed by postoperative adjuvant therapy. BRFS, bladder recurrence-free survival; DFS, disease-free survival; CFS, cystectomy-free survival; DMFS, distant metastasis-free survival

The estimated 1-year and 2-year BRFS rates were 86.50% (95% CI 63.7–95.45%) and 78.64% (95% CI 50.79–91.83%) respectively. Of these, 18 patients (85.71%) had a BRFS ≥ 12 months and 14 (66.67%) had a BRFS ≥ 24 months. The 2-year CFS and 2-year DFS rates were 83.00% (95% CI 53.53–94.59%) and 73.39% (95% CI 46.14–88.36%), respectively. For stage T1 patients (n = 16), the 2-year DMFS rate was 95.45% (95% CI 71.87–99.34%; Table 2 and Fig. 1).

**Table 2:** Efficacy outcomes

Variables	All patients (n = 21) (%, 95% CI)
1-year BRFS	86.50 (63.7–95.45)
2-year BRFS	78.64 (50.79–91.83)
2-year CFS	83.00 (53.53–94.59)
2-year DFS	73.39 (46.14–88.36)
2-year DMFS for cT1 (n = 16)	95.45 (71.87–99.34)

BRFS, bladder recurrence-free survival; DFS, disease-free survival; CFS, cystectomy-free survival; DMFS, distant metastasis-free survival

### Safety

In terms of safety (Table 3), a total of 14 (66.67%) patients experienced at least one treatment-related AEs (TRAEs). The most frequent TRAEs of any grade included nausea (6/21, 28.57%), skin rash (2/21, 9.52%), and urinary frequency (2/21, 9.52%). Most TRAEs were classified as grade 1–2 (19, 90.48%), while 2 patients (9.52%) had grade 3–4. The grade ≥ 3 TRAEs included were hypophysitis (1/21, 4.76%) and myasthenia (1/21, 4.76%).

**Table 3:** Adverse events

Adverse event	Patients, No., (%) (n = 21)	
	Any grade	Grade 3 or 4
Any treatment-related		
Nausea	6 (28.57)	0
Skin rash	2 (9.52)	0
Urinary frequency	2 (9.52)	0
Dizziness	1 (4.76)	0
Cystitis	1 (4.76)	0
Creatinine increased	1 (4.76)	0
Renal insufficiency	1 (4.76)	0
Hypokalemia	1 (4.76)	0
Hypothyroidism	1 (4.76)	0
Hypophysitis	1 (4.76)	1 (4.76)
Pneumonitis	1 (4.76)	0
Myasthenia	1 (4.76)	1 (4.76)
Any immune-related		
Urinary frequency	2 (9.52)	0
Skin rash	2 (9.52)	0
Pneumonitis	1 (4.76)	0
Hypophysitis	1 (4.76)	1 (4.76)
Myasthenia gravis	1 (4.76)	1 (4.76)

Seven patients (33.33%) had immune-related AEs (iRAEs), which the most frequent were urinary frequency (9.52%) and skin rash (9.52%). The grade 3 irAEs were observed in 2 patients (9.52%) including hypophysitis (4.76%) and myasthenia gravis (4.76%). There was 1 death that was not associated with treatment.

## Discussion

To our knowledge, this is the first study to evaluate the application of tislelizumab-based combination regimen in bladder-sparing strategies against HR-NMIBC. Overall, the combination regimen of tislelizumab plus BCG with or without chemotherapy yields promising clinical outcomes with 1-year BRFS rate of 86.50%, 2-year BRFS rate of 78.64%, 2-year CFS rate of 83.00%, and 2-year DFS of 73.39%. In addition, the safety profile of this regimen is tolerable and manageable. This regimen may be considered a clinically active non-surgical treatment option in this difficult-to-treat population.

HR-NMIBC is a difficult-to-treat disease with few approved therapeutic options and its management is one of the most challenging issues in the urological community [2]. TURBT followed by intravesical instillations with BCG is recommended by guidelines from European Association of Urology and American Society of Urologic Oncology for HR-NMIBC patients who have strong desire for bladder-sparing treatments or have comorbidities that preclude them from RC [16, 17]. However there are several contemporary problems with the use of BCG, such as high recurrence rates of 80% for patients who receive BCG, up to 45% of patients develop MIBC within 5 years, and ongoing BCG shortages in many countries raise concerns [18]. An accumulating body of evidence suggests that in the era of immunotherapy, high tumor mutational burden (TMB), DNA damage-response mutations, the presence of genomic instability, and high PD-L1 expression make bladder cancer suitable for immunotherapy [19]. The spotlight on immunotherapeutic strategies for bladder cancer is intensifying. In the KEYNOTE-057 trial, pembrolizumab monotherapy has shown promising results in the treatment of high-risk BCG-unresponsive NMIBC, with a complete response rate (CR) of 41% at 3 months after a median follow-up 36.4 months [7]. Consequently, pembrolizumab has received approval for the treatment of BCG-unresponsive NMIBC patients. Another study similarly reported a favorable CR rate of 41.7% at 6 months with atezolizumab monotherapy in BCG-unresponsive NMIBC patients [20].

Studies have shown that PD-L1 and PD-1 expression is associated with BCG immune-resistance [10, 21]. BCG instillation seems to induce the expression of PD-L1 in tumor and inflammatory cells through the induction of CD8^+^ T cells, which are the major responsible for IFN-γ (which is associated to a BCG-unresponsive state) production [22]. Pierconti et al. found that PD-L1 expression in tumor cells and in immune cells was higher in BCG-unresponsive bladder cancer patients than in BCG-responders, suggesting BCG itself could enhance PD-1 and PD-L1 [23]. This provides the grounds for trials that are testing anti-PD-1 or anti-PD-L1 antibodies in association with BCG as front-line therapy in NMIBC in BCG-naïve patients or in patients not reaching a CR after BCG induction [10, 21]. Inman et al. conducted a Phase 1b/2 study investigating the use of atezolizumab with or without BCG instillation in HR-NMIBC, and found a monotherapy CR rate of 33.3%, while the combination group achieved a CR rate of 42% [24]. Our study similarly supports this conclusion, indicating that the addition of immunotherapeutic agents to conventional treatment modalities can yield effective outcomes, reducing recurrence and progression. In our study, we observed a promising inhibition of disease progression and recurrence, with 1-year BRFS rate of 86.50%, a 2-year BRFS rate of 78.64%, and a 2-year DFS of 73.39%, which underscored the potential benefits of utilizing immunotherapeutic in the management of HR-NMIBC. Furthermore, for stage T1 patients, GC chemotherapy was performed to enhance the management of disease metastasis and recurrence. Chu et al. have revealed that patients with pathologic features of high grade T1 disease harbored more *TP53, ERBB2/Her2,* and *RB1* mutations and a genomic profile more similar to T2 tumors than NMIBC, suggesting an increased risk of infiltration and metastasis [25]; Meanwhile, NCCN guidelines show that chemotherapy given after TURBT may prevent tumor cell implantation and recurrence in select subgroups of patients [26]. In addition, studies are demonstrating promising outcomes with single or combination chemotherapy agents (such as sequential intravesical gemcitabine and docetaxel, gemcitabine bladder instillation) in the treatment of HR-NMIBC, which has been shown to confer approximately 2-year BRFS of 57% [27] 2-year DFS of 30–40% [18, 28], and 2-year CFS of 79–86% [27, 29]. In comparison, our study shows improved results, particularly with numerically higher rate for 2-year BRFS (78.64%) and 2-year DFS (73.39%); additionally, our study stabilizes 2-year CFS at 83%. However, it is important to note that the majority of the aforementioned evaluations of chemotherapy treatment for HR-NMIBC stem from smaller series studies and retrospective analyses, necessitating careful consideration when extrapolating these findings.

Overall, compared with the above studies, the results in our study demonstrated the potential efficacy of this combination therapy of tislelizumab and BCG with or without chemotherapy, with a notable 76.2% of patients achieving disease stability, accompanied by a marked inhibition of recurrence. Notably, key outcome measures such as BRFS, CFS, and DFS reached a plateau with a prolonged “tail” approximately 2 years post-treatment initiation. In particular, is the behavior of DMFS, which not only entered a stable phase earlier than other parameters but has also consistently maintained this stable status. Although OS remains the gold standard for assessing oncological efficacy, the observation period for mature OS outcomes is often protracted [30]. Studies indicate that for bladder cancer patients undergoing platinum-based adjuvant chemotherapy, DMFS may serve as a potentially crucial surrogate endpoint for OS [31]. These findings in our study collectively emphasize the potential viability of the combination strategy involving tislelizumab and BCG with or without chemotherapy in HR-NMIBC, offering additional choices in the spectrum of treatment options for this patient cohort.

It is critical to consider the safety profile when patients receive potentially effective drug combination regimens. Overall, the tislelizumab combination regimen is generally well tolerated and manageable in our study. The TRAEs of any grade in our study was 66.67%, with grade ≥ 3 TRAEs occurring in 9.52%. Notably, the incidence of grade ≥ 3 TRAEs is lower than observed in other PD-1 studies, such as the Keynote 057 study (13%) and the SWOG S1605 study (approximately 17%) [7, 20]. The increased incidence of AEs is associated with chemotherapy-related factors such as nausea, vomiting, and renal dysfunction. Throughout the course of patient treatment, a multidisciplinary team (MDT) managed adverse reactions comprehensively, which included specialists from oncology, hematology, nephrology, gastroenterology, etc. Early detection and timely intervention by the MDT ensured patient safety.

Certain limitations of our study should be acknowledged. Firstly, our study is limited by its retrospective nature, hence, there exists a potential selection bias. Secondly, the sample size of this study was relatively small. Future studies should include a larger sample size, and randomized clinical studies should be conducted. Thirdly, there is a lack of long-term follow-up results. Long-term follow-up is needed to understand the long-term prognosis of patients. Finally, there is no uniform treatment protocol in our study, but some patients with a strong desire to preserve their bladder may intolerant to cisplatin-based chemotherapy based on clinical reality. There is a need to extend the sample size to classify patients into subgroups (with or without chemotherapy) in order to validate these preliminary findings and to better explore the optimal treatment options.

## Conclusion

In summary, this retrospective study investigated the efficacy and safety of tislelizumab plus BCG with or without chemotherapy as a bladder-sparing treatment for HR-NMIBC. The results of this study had meaningful clinical activity and a manageable safety profile, suggesting this regimen is feasible and safe. These findings offer a fresh perspective on treatment strategies for HR-NMIBC. In the future, it is essential to explore the therapeutic effects of this regimen on HR-NMIBC through larger-scale randomized controlled studies.

## Data Availability

The datasets used and/or analyzed during the current study are available from the corresponding author upon reasonable request.

## References

[1] van den Bosch S, Alfred WJ (2011). Long-term cancer-specific survival in patients with high-risk, non–muscle-invasive bladder cancer and tumour progression: a systematic review. Eur Urol.

[2] Compérat E, Amin MB, Cathomas R, Choudhury A, De Santis M, Kamat A (2022). Current best practice for bladder cancer: a narrative review of diagnostics and treatments. Lancet.

[3] Oddens J, Brausi M, Sylvester R, Bono A, van de Beek C, van Andel G (2013). Final results of an EORTC-GU cancers group randomized study of maintenance bacillus Calmette-Guérin in intermediate- and high-risk Ta, T1 papillary carcinoma of the urinary bladder: one-third dose versus full dose and 1 year versus 3 years of maintenance. Eur Urol.

[4] Hussain MH, Wood DP, Bajorin DF, Bochner BH, Dreicer R, Lamm DL (2009). Bladder cancer: narrowing the gap between evidence and practice. J Clin Oncol.

[5] Shabsigh A, Korets R, Vora KC, Brooks CM, Cronin AM, Savage C (2009). Defining early morbidity of radical cystectomy for patients with bladder cancer using a standardized reporting methodology. Eur Urol.

[6] Alfred Witjes J, Lebret T, Compérat EM, Cowan NC, De Santis M, Bruins HM (2017). Updated 2016 EAU guidelines on muscle-invasive and metastatic bladder cancer. Eur Urol.

[7] Balar AV, Kamat AM, Kulkarni GS, Uchio EM, Boormans JL, Roumiguié M (2021). Pembrolizumab monotherapy for the treatment of high-risk non-muscle-invasive bladder cancer unresponsive to BCG (KEYNOTE-057): an open-label, single-arm, multicentre, phase 2 study. Lancet Oncol.

[8] Hato SV, Khong A, de Vries IJ, Lesterhuis WJ (2014). Molecular pathways: the immunogenic effects of platinum-based chemotherapeutics. Clin Cancer Res.

[9] de Jong FC, Rutten VC (2021). Improving Anti-PD-1/PD-L1 therapy for localized bladder cancer. Int J Mol Sci.

[10] Audisio A, Buttigliero C, Delcuratolo MD, Parlagreco E, Audisio M, Ungaro A (2022). New perspectives in the medical treatment of non-muscle-invasive bladder cancer: immune checkpoint inhibitors and beyond. Cells.

[11] Lee A, Keam SJ (2020). Tislelizumab: first approval. Drugs.

[12] Zhang T, Song X, Xu L, Ma J, Zhang Y, Gong W (2018). The binding of an anti-PD-1 antibody to FcγRΙ has a profound impact on its biological functions. Cancer Immunol Immunother.

[13] Feng Y, Hong Y, Sun H, Zhang B, Wu H, Li K (2019). Abstract 2383: the molecular binding mechanism of tislelizumab, an investigational anti-PD-1 antibody, is differentiated from pembrolizumab and nivolumab. Can Res.

[14] Xu J, Bai Y, Xu N, Li E, Wang B, Wang J (2020). Tislelizumab plus chemotherapy as first-line treatment for advanced esophageal squamous cell carcinoma and gastric/gastroesophageal junction adenocarcinoma. Clin Cancer Res.

[15] Wang J, Lu S, Yu X, Hu Y, Sun Y, Wang Z (2021). Tislelizumab plus chemotherapy vs chemotherapy alone as first-line treatment for advanced squamous non-small-cell lung cancer: a phase 3 randomized clinical trial. JAMA Oncol.

[16] Chang SS, Boorjian SA, Chou R, Clark PE, Daneshmand S, Konety BR (2016). Diagnosis and treatment of non-muscle invasive bladder cancer: AUA/SUO guideline. J Urol.

[17] Babjuk M, Burger M, Compérat EM, Gontero P, Mostafid AH, Palou J (2019). European association of urology guidelines on non-muscle-invasive bladder cancer (TaT1 and carcinoma in situ)—2019 update. Eur Urol.

[18] Packiam VT, Werntz RP, Steinberg GD (2019). Current clinical trials in non-muscle-invasive bladder cancer: heightened need in an era of chronic BCG shortage. Curr Urol Rep.

[19] Rani B, Ignatz-Hoover JJ, Rana PS (2023). Current and emerging strategies to treat urothelial carcinoma. Cancers.

[20] Black PC, Tangen CM, Singh P, McConkey DJ, Lucia MS, Lowrance WT (2023). Phase 2 trial of atezolizumab in bacillus Calmette-Guérin-unresponsive high-risk non-muscle-invasive bladder cancer: SWOG S1605. Eur Urol.

[21] Kates M, Matoso A, Choi W, Baras AS, Daniels MJ, Lombardo K (2020). Adaptive immune resistance to intravesical BCG in non-muscle invasive bladder cancer: implications for prospective BCG-unresponsive trials. Clin Cancer Res.

[22] Bellmunt J, Mullane SA, Werner L, Fay AP, Callea M, Leow JJ (2015). Association of PD-L1 expression on tumor-infiltrating mononuclear cells and overall survival in patients with urothelial carcinoma. Ann Oncol.

[23] Pierconti F, Raspollini MR, Martini M, Larocca LM, Bassi PF, Bientinesi R (2020). PD-L1 expression in bladder primary in situ urothelial carcinoma: evaluation in BCG-unresponsive patients and BCG responders. Virchows Arch.

[24] Inman BA, Hahn NM, Stratton K, Kopp R, Sankin A, Skinner E (2023). A phase 1b/2 study of Atezolizumab with or without Bacille Calmette-Guérin in patients with high-risk non-muscle-invasive bladder cancer. Eur Urol Oncol.

[25] Chu C, Chen J, Escano MDJ, Whiting K, Ostrovnaya I, Jiang S (2023). MP63-08 clinical and genomic risk features of high Grade T1 non-muscle invasive bladder cancer. J Urol.

[26] Flaig TW, Spiess PE, Agarwal N, Bangs R, Boorjian SA, Buyyounouski MK (2020). Bladder cancer, version 3.2020, NCCN clinical practice guidelines in oncology. J Natl Compr Cancer Network.

[27] Vila-Reyes H, Decastro GJ, Faiena I, Pak JS, Lee K, Li G (2020). MP73–09 Effectiveness of combination cisplatin-based intravesical chemotherapy for BCG-refractory bladder cancer. J Urol.

[28] Milbar N, Kates M, Chappidi MR, Pederzoli F, Yoshida T, Sankin A (2017). Oncological outcomes of sequential intravesical gemcitabine and docetaxel in patients with non-muscle invasive bladder cancer. Bladder Cancer.

[29] Yim K, Melnick K, Mott SL, Carvalho FLF, Zafar A, Clinton TN (2023). Sequential intravesical gemcitabine/docetaxel provides a durable remission in recurrent high-risk NMIBC following BCG therapy. Urol Oncol Semin Orig Investig.

[30] Driscoll JJ, Rixe O (2009). Overall survival: still the gold standard: why overall survival remains the definitive end point in cancer clinical trials. Cancer J.

[31] Sternberg C, Squifflet P, Burdett S, Fisher D, Saad ED, Kurt M (2022). 1746P disease-free survival (DFS) and distant metastasis-free survival (DMFS) as surrogates for overall survival (OS) in adjuvant treatment of muscle-invasive bladder cancer (MIBC). Ann Oncol.

